# Impact of long-term white matter hyperintensity changes on mobility and dexterity

**DOI:** 10.1093/braincomms/fcae133

**Published:** 2024-05-07

**Authors:** Angela C C Jochems, Susana Muñoz Maniega, Francesca M Chappell, Una Clancy, Carmen Arteaga, Daniela Jaime Garcia, Olivia K L Hamilton, Will Hewins, Rachel Locherty, Ellen V Backhouse, Gayle Barclay, Charlotte Jardine, Donna McIntyre, Iona Gerrish, Yajun Cheng, Xiaodi Liu, Junfang Zhang, Agniete Kampaite, Eleni Sakka, Maria Valdés Hernández, Stewart Wiseman, Michael S Stringer, Michael J Thrippleton, Fergus N Doubal, Joanna M Wardlaw

**Affiliations:** Centre for Clinical Brain Sciences, University of Edinburgh, EH16 4SB Edinburgh, United Kingdom; MRC UK Dementia Research Institute at the University of Edinburgh, EH16 4SB Edinburgh, United Kingdom; Centre for Clinical Brain Sciences, University of Edinburgh, EH16 4SB Edinburgh, United Kingdom; MRC UK Dementia Research Institute at the University of Edinburgh, EH16 4SB Edinburgh, United Kingdom; Centre for Clinical Brain Sciences, University of Edinburgh, EH16 4SB Edinburgh, United Kingdom; MRC UK Dementia Research Institute at the University of Edinburgh, EH16 4SB Edinburgh, United Kingdom; Centre for Clinical Brain Sciences, University of Edinburgh, EH16 4SB Edinburgh, United Kingdom; MRC UK Dementia Research Institute at the University of Edinburgh, EH16 4SB Edinburgh, United Kingdom; Centre for Clinical Brain Sciences, University of Edinburgh, EH16 4SB Edinburgh, United Kingdom; MRC UK Dementia Research Institute at the University of Edinburgh, EH16 4SB Edinburgh, United Kingdom; Centre for Clinical Brain Sciences, University of Edinburgh, EH16 4SB Edinburgh, United Kingdom; MRC UK Dementia Research Institute at the University of Edinburgh, EH16 4SB Edinburgh, United Kingdom; Centre for Clinical Brain Sciences, University of Edinburgh, EH16 4SB Edinburgh, United Kingdom; MRC UK Dementia Research Institute at the University of Edinburgh, EH16 4SB Edinburgh, United Kingdom; MRC/CSO Social and Public Health Sciences Unit, School of Health and Wellbeing, University of Glasgow, G12 8TB Glasgow, United Kingdom; Centre for Clinical Brain Sciences, University of Edinburgh, EH16 4SB Edinburgh, United Kingdom; MRC UK Dementia Research Institute at the University of Edinburgh, EH16 4SB Edinburgh, United Kingdom; Centre for Clinical Brain Sciences, University of Edinburgh, EH16 4SB Edinburgh, United Kingdom; MRC UK Dementia Research Institute at the University of Edinburgh, EH16 4SB Edinburgh, United Kingdom; Centre for Clinical Brain Sciences, University of Edinburgh, EH16 4SB Edinburgh, United Kingdom; MRC UK Dementia Research Institute at the University of Edinburgh, EH16 4SB Edinburgh, United Kingdom; Centre for Clinical Brain Sciences, University of Edinburgh, EH16 4SB Edinburgh, United Kingdom; Edinburgh Imaging Facility, Royal Infirmary of Edinburgh, EH16 4TJ Edinburgh, United Kingdom; Centre for Clinical Brain Sciences, University of Edinburgh, EH16 4SB Edinburgh, United Kingdom; Edinburgh Imaging Facility, Royal Infirmary of Edinburgh, EH16 4TJ Edinburgh, United Kingdom; Centre for Clinical Brain Sciences, University of Edinburgh, EH16 4SB Edinburgh, United Kingdom; Edinburgh Imaging Facility, Royal Infirmary of Edinburgh, EH16 4TJ Edinburgh, United Kingdom; Centre for Clinical Brain Sciences, University of Edinburgh, EH16 4SB Edinburgh, United Kingdom; Edinburgh Imaging Facility, Royal Infirmary of Edinburgh, EH16 4TJ Edinburgh, United Kingdom; Centre for Clinical Brain Sciences, University of Edinburgh, EH16 4SB Edinburgh, United Kingdom; MRC UK Dementia Research Institute at the University of Edinburgh, EH16 4SB Edinburgh, United Kingdom; Department of Neurology, West China Hospital of Sichuan University, 610041 Chengdu, China; Centre for Clinical Brain Sciences, University of Edinburgh, EH16 4SB Edinburgh, United Kingdom; MRC UK Dementia Research Institute at the University of Edinburgh, EH16 4SB Edinburgh, United Kingdom; Department of Medicine, LKS Faculty of Medicine, University of Hong Kong, Hong Kong, China; Centre for Clinical Brain Sciences, University of Edinburgh, EH16 4SB Edinburgh, United Kingdom; MRC UK Dementia Research Institute at the University of Edinburgh, EH16 4SB Edinburgh, United Kingdom; Department of Neurology, Shanghai General Hospital, Shanghai Jiao Tong University School of medicine, 200080 Shanghai, China; Centre for Clinical Brain Sciences, University of Edinburgh, EH16 4SB Edinburgh, United Kingdom; Centre for Clinical Brain Sciences, University of Edinburgh, EH16 4SB Edinburgh, United Kingdom; Centre for Clinical Brain Sciences, University of Edinburgh, EH16 4SB Edinburgh, United Kingdom; MRC UK Dementia Research Institute at the University of Edinburgh, EH16 4SB Edinburgh, United Kingdom; Centre for Clinical Brain Sciences, University of Edinburgh, EH16 4SB Edinburgh, United Kingdom; MRC UK Dementia Research Institute at the University of Edinburgh, EH16 4SB Edinburgh, United Kingdom; Centre for Clinical Brain Sciences, University of Edinburgh, EH16 4SB Edinburgh, United Kingdom; MRC UK Dementia Research Institute at the University of Edinburgh, EH16 4SB Edinburgh, United Kingdom; Centre for Clinical Brain Sciences, University of Edinburgh, EH16 4SB Edinburgh, United Kingdom; MRC UK Dementia Research Institute at the University of Edinburgh, EH16 4SB Edinburgh, United Kingdom; Edinburgh Imaging Facility, Royal Infirmary of Edinburgh, EH16 4TJ Edinburgh, United Kingdom; Centre for Clinical Brain Sciences, University of Edinburgh, EH16 4SB Edinburgh, United Kingdom; MRC UK Dementia Research Institute at the University of Edinburgh, EH16 4SB Edinburgh, United Kingdom; Centre for Clinical Brain Sciences, University of Edinburgh, EH16 4SB Edinburgh, United Kingdom; MRC UK Dementia Research Institute at the University of Edinburgh, EH16 4SB Edinburgh, United Kingdom; Edinburgh Imaging Facility, Royal Infirmary of Edinburgh, EH16 4TJ Edinburgh, United Kingdom

**Keywords:** mobility, dexterity, white matter hyperintensity, imaging, small vessel disease

## Abstract

White matter hyperintensities (WMH), a common feature of cerebral small vessel disease, are related to worse clinical outcomes after stroke. We assessed the impact of white matter hyperintensity changes over 1 year after minor stroke on change in mobility and dexterity, including differences between the dominant and non-dominant hands and objective in-person assessment versus patient-reported experience. We recruited participants with lacunar or minor cortical ischaemic stroke, performed medical and cognitive assessments and brain MRI at presentation and at 1 year. At both time points, we used the timed-up and go test and the 9-hole peg test to assess mobility and dexterity. At 1 year, participants completed the Stroke Impact Scale. We ran two linear mixed models to assess change in timed-up and go and 9-hole peg test, adjusted for age, sex, stroke severity (National Institutes of Health Stroke Scale), dependency (modified Rankin Score), vascular risk factor score, white matter hyperintensity volume (as % intracranial volume) and additionally for 9-hole peg test: Montreal cognitive assessment, hand (dominant/non-dominant), National Adult Reading Test (premorbid IQ), index lesion side. We performed ordinal logistic regression, corrected for age and sex, to assess relations between timed-up and go and Stroke Impact Scale mobility, and 9-hole peg test and Stroke Impact Scale hand function. We included 229 participants, mean age 65.9 (standard deviation = 11.13); 66% male. 215/229 attended 1-year follow-up. Over 1 year, timed-up and go time increased with aging (standardized β [standardized 95% Confidence Interval]: 0.124[0.011, 0.238]), increasing National Institutes of Health Stroke Scale (0.106[0.032, 0.180]), increasing modified Rankin Score (0.152[0.073, 0.231]) and increasing white matter hyperintensity volume (0.176[0.061, 0.291]). Men were faster than women (−0.306[0.011, 0.238]). Over 1 year, slower 9-hole peg test was related to use of non-dominant hand (0.290[0.155, 0.424]), aging (0.102[0.012, 0.192]), male sex (0.182[0.008, 0.356]), increasing National Institutes of Health Stroke Scale (0.160 [0.094, 0.226]), increasing modified Rankin Score (0.100[0.032, 0.169]), decreasing Montreal cognitive assessment score (−0.090[−0.167, −0.014]) and increasing white matter hyperintensity volume (0.104[0.015, 0.193]). One year post-stroke, Stroke Impact Scale mobility worsened per second increase on timed-up and go, odds ratio 0.67 [95% confidence interval 0.60, 0.75]. Stroke Impact Scale hand function worsened per second increase on the 9-hole peg test for the dominant hand (odds ratio 0.79 [0.71, 0.86]) and for the non-dominant hand (odds ratio 0.88 [0.83, 0.93]). Decline in mobility and dexterity is associated with white matter hyperintensity volume increase, independently of stroke severity. Mobility and dexterity declined more gradually for stable and regressing white matter hyperintensity volume. Dominant and non-dominant hands might be affected differently. In-person measures of dexterity and mobility are associated with self-reported experience 1-year post-stroke.

## Teaser

Jochems *et al.* report that mobility and dexterity are affected by white matter hyperintensity volume longitudinal change after minor ischaemic stroke, independently of stroke severity. Dominant and non-dominant hands might be differently affected. In-person measures of dexterity and mobility are associated with self-reported experience 1 year after stroke.

## Graphical Abstract

**Graphical abstract:**
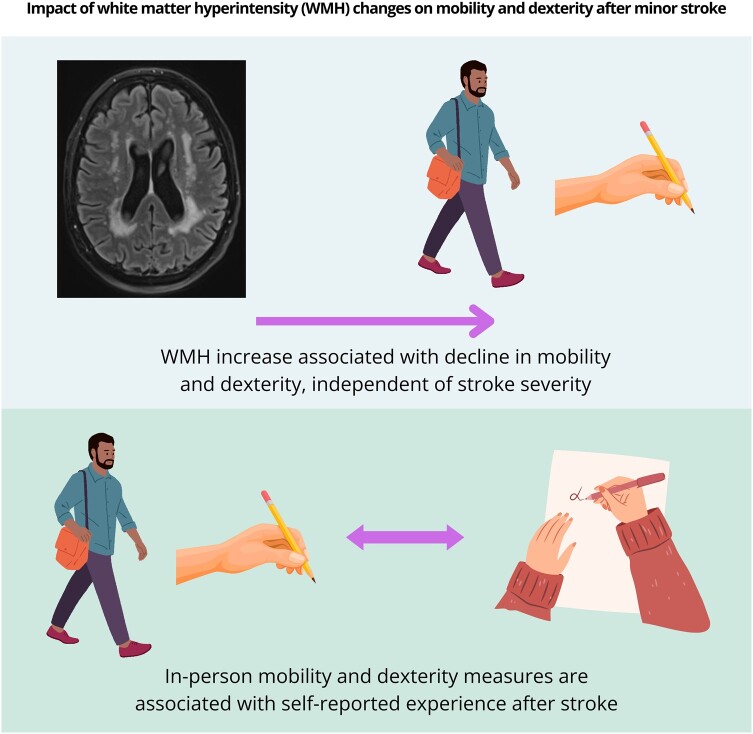

## Introduction

Cerebral small vessel disease (SVD) is a disease of the small perforating blood vessels in the brain. SVD features appear on neuroimaging as subcortical infarcts, lacunes, perivascular spaces, microbleeds and white matter hyperintensities (WMH) of presumed vascular origin.^1^ WMH are known to progress over time and a recent systematic review and meta-analysis (*n =* 12 284) showed that WMH can also regress.^2^

SVD is the most common cause of vascular cognitive impairment and is related to an increased risk of stroke, dementia and death.^3^ SVD is also associated with worse clinical outcomes after acute stroke. In community-dwelling people, SVD features, and in particular WMH, have been associated with impaired mobility^4-12^ and dexterity.^5,10,13^ Gait, mobility^14^ and dexterity^15^ are commonly affected after stroke and can negatively affect quality of life,^16^ daily functioning,^17^ participation and reintegration into the community after stroke.^16,18-20^ Worse fine motor dexterity 3 months after stroke is also related to worse cognitive performance.^21^ However, for both mobility and dexterity, most evidence comes from studies with small sample sizes^17,18^ and patients with severe stroke^16^ or varying stroke severities.^19,21^ Despite the clear links between worsening mobility and dexterity after stroke and WMH in community-dwelling populations, little is known about any additional contribution of WMH to mobility and dexterity outcomes after minor lacunar and cortical ischaemic stroke. Patients with minor strokes might have less apparent physical symptoms that could easily go unnoticed. A previous study showed that mobility and hand function might still be affected 3 years after minor stroke^22^ and that WMH might have an added effect on impaired hand function after stroke.^1,23^

We aimed to document the impact of long-term WMH changes on mobility and dexterity over 1 year after a minor ischaemic stroke, as these lesions might have an additive effect after the original stroke. Additionally, we explore whether the dominant and non-dominant hands are affected differently. We also compare objective in-person assessments of mobility and dexterity with a self-reported questionnaire to assess whether objective measures are representative of the experience of patients.

## Materials and methods

### Participants

Participants were included in the Mild Stroke Study 3,^24^ a prospective cohort study of patients presenting to the Lothian Stroke Services. Patients were included if they were ≥18 years old and had an acute minor ischaemic stroke (minor defined as National Institute of Health Stroke Scale (NIHSS)^25^ < 8 and independence in daily living, i.e. a modified Rankin scale (mRS)^26^ of ≤2) at time of recruitment. In the case of hospital admission, participants might have been seen by a physiotherapist and occupational therapist. However, they did not receive prolonged neurorehabilitation. No participants were in-patients at time of recruitment. Participants were excluded if they had MRI contraindications, severe respiratory, cardiac or neurological conditions. The final stroke diagnosis was made based on symptom classification, as described previously,^27^ supplemented by additional relevant investigations and diagnostic CT or MRI at time of presenting to stroke services. All participants provided written informed consent. Ethical approval was granted by Southeast Scotland Regional Ethics Committee (reference 18/SS/0044).

Participants attended the baseline visit within 3 months after stroke. They underwent brain MRI, medical, cognitive, mobility and dexterity assessments. As part of the medical assessment, we collected their medical history to establish vascular risk factors including hypertension, hypercholesterolemia, diabetes mellitus and history of smoking. All participants were invited for a follow-up visit 1 year later for medical, cognitive, mobility and dexterity assessments, and underwent further brain MRI.

### Assessments

At the baseline visit, we assessed stroke severity with the NIHSS and dependency with the mRS. NIHSS scores range from 0 to 42 with higher scores indicating greater severity. The mRS ranges from 0 (no symptoms) to 6 (death), with scores below two indicating independence in activities of daily living, even when symptoms are present. Higher scores indicate greater dependency.

Global cognition was assessed with the Montreal Cognitive Assessment (MoCA)^28^ at both visits. Alternative versions of the MoCA were used at subsequent visits to minimize learning effects.^29^ The MoCA is a cognitive screening test widely used to assess cognitive impairment after stroke, covering several cognitive domains.^30^ Scores range from 0 to 30, with higher scores related to better cognition. Scores below 26 might indicate cognitive impairment.^28^ The National Adult Reading Test (NART)^31^ requires participants to read out a list of 50 irregular English words. Vocabulary knowledge is stable over time and can measure peak cognitive ability^32^ and is used to establish pre-morbid intelligence.^33^ The pronunciation of these irregular words can be maintained after stroke.^34^

Mobility was assessed in-person with the Timed Up and Go (TUG) test.^35,36^ Participants were asked to stand up from a chair, walk 3 m, turn around, walk back and sit back down on the chair. We recorded the time (seconds) it took to complete the task. Dexterity was recorded with the 9 Hole Peg Test (9HPT).^37,38^ Participants were asked to place nine pegs, as quickly as possible into nine holes in a board. We recorded the time it takes to complete the task for each hand. We maintained a cut-off of 50 sec.^39^ Scores were identified separately for the dominant and non-dominant hand.

At the 1-year visit, we repeated the NIHSS, mRS, MoCA, TUG and 9HPT. Before the in-person study visit, participants were asked to fill in the Stroke Impact Scale (SIS)^40^ by post. The SIS is a questionnaire that evaluates how the stroke has impacted the patient’s life. It covers eight domains: strength in the extremities most affected by the stroke, memory and thinking, emotionality, communication, activities of daily living, mobility, hand function in the hand most affected by stroke, and societal participation, and assesses recovery after stroke on a scale from 0 (no recovery) to 100 (full recovery). In this analysis, we only focussed on the domains of mobility and hand function. For the mobility (scores range from 9 to 45) and hand function domains (ranging scores from 5 to 25), with lower scores indicating worse impact on life.

### Imaging acquisition

The full details of the Mild Stroke Study three brain imaging protocols are published.^24^ Briefly, at both visits participants underwent brain MRI on the same 3T scanner (Siemens Prisma, Erlangen, Germany) with the same sequences. The images were acquired using a 32-channel head coil (Siemens Healthcare, Erlangen, Germany). The MRI protocol included 3D T1-weighted (1 mm^3^ isotropic; TR/TE/TI = 2500/4.37/1100 ms), T2-weighted (0.9 mm^3^ isotropic; TR/TE = 3200/408 ms), FLAIR (1 mm^3^ isotropic; TR/TE/TI = 5000/388/1800 ms), diffusion-tensor imaging (64 diffusion-weighted images (DWI) with *b* = 1000 s/mm^2^; 2 mm^3^ isotropic voxels TR/TE = 4300/74 ms) and 3D proton density (PD) imaging (1.2 mm^3^ isotropic resolution, TR/TE = 865/1.82 ms). The MRI is monitored with a quality assurance programme to check for scanner performance issues and to maintain consistent scanner function and image quality.

### Imaging processing and analysis

The image processing protocol is described elsewhere.^24^ All image sequences were analysed using validated computational pipelines and were co-registered to the first visit T2-weighted image using FLIRT^41^ from FSL.^42^ The intracranial volumes (ICV) were generated computationally from the PD image, and they were checked and manually edited if necessary. WMH were defined according to STRIVE criteria.^1^ Details on the WMH segmentation and associated pipeline are published elsewhere.^43^ The pipelines were developed in the course of previous similar studies^44,45^ and further refined. We acquired WMH volumes (mm^3^) from FLAIR images and removed false positives in the vicinities of the choroid plexus, aqueduct, third and fourth ventricles, using Freesurfer (https://surfer.nmr.mgh.harvard.edu/). To further exclude any hyperintensities that unlikely reflect pathology, a lesion distribution probabilistic template was applied to the thresholded images,^46^ and the result was visually checked and manually corrected if still necessary. WMH volumes were normalized for the ICV (mm^3^) to account for participant’s head size and reported as a percentage of ICV (%ICV) unless otherwise stated.

**Figure 1:**
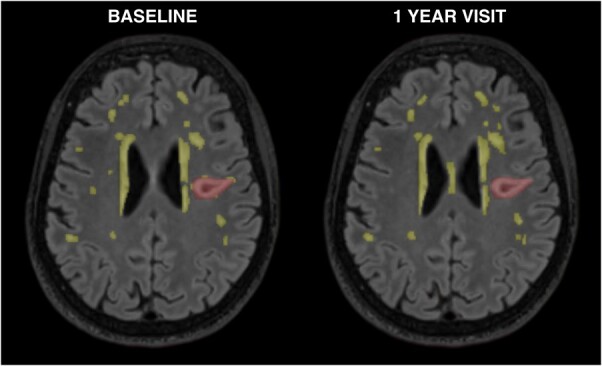
**Segmented WMH and index stroke at baseline and 1 year.** Example of baseline and 1 year scans of one participant (age 66 at baseline) with WMH segmented (yellow) and index stroke lesion (red). WMH: White matter hyperintensity.

Old and acute stroke lesions were manually drawn on the FLAIR sequence by an experienced image analyst, guided by other MRI sequences. All cases were discussed with a neuroradiologist and the masks were adjusted if required. Stroke lesions were identified at both visits and excluded from the WMH volumes to avoid erroneous measures of WMH volume. See Fig. 1 for an example of segmented WMH and index stroke lesion at baseline and 1 year. Inter-rater and intra-rater variability in manually rectifying abnormal tissues and ICV have been analysed and reported previously.^44^

The side of the index lesion was recorded as part of visual MRI assessment. Locations of index lesion include left hemisphere, right hemisphere, both hemispheres, or no visible lesion when no index lesion was visible. All cases were checked by a neuroradiologist (JMW).

### Statistical analysis

All statistical analyses were performed with R Studio, using R version 4.2.2^47^ and the ggplot2, lmer, emmeans and MASS packages. To assess longitudinal impact of WMH changes over 1 year we performed repeated measurements linear mixed models for which we reported standardized betas and not the original units of the variables to allow us to compare the influence of the variables. There were no gross violations of the assumptions for all statistical models. We also calculated quintiles of WMH volume change. Volume change, as used in the quintile plots, was defined as the difference in WMH volume between the 1 year visit and the baseline visit.

We performed two repeated measures linear mixed models with TUG and 9HPT as outcomes. The models included a random intercept for the individual participants to account for individual differences in TUG and 9HPT. We included predictors based on relevance to our research question and on the available literature, not on statistical significance. For both models, predictors included age (in years, centred to age at baseline)^48^ and sex,^38,49^ NIHSS, mRS, combined vascular risk factor (VRF) score, WMH volume. We used a combined VRF score which summarizes the presence (yes or no) of hypertension, hypercholesterolemia, diabetes mellitus and smoking (current or ex-smoker ≤1 year) and ranges from 0 (no vascular risk factors present) to 4 (all vascular risk factors present).^50^

For the 9HPT model, we also added a random slope for hand, i.e. whether the dominant or non-dominant hand was used for the test, to allow for differences in the slope for dominant and non-dominant hand. We also added the following predictors: hand (dominant and non-dominant), side of brain of index lesion, MoCA and NART.^51,52^ Examples of the linear mixed models can be found in the Supplementary material. We obtained the estimated marginal means for handedness (dominant versus non-dominant hand), using the same linear mixed model, to examine any differences in effect between the dominant and non-dominant hand. For both the TUG and 9HPT models, we examined the role of stroke subtype to see whether a cortical or subcortical stroke might be relevant for the outcomes. To further explore whether predictors differently affect the 9HPT scores for the dominant and non-dominant hand, we performed linear mixed models with an interaction term between hand and the predictors: age, sex, NIHSS, mRS, VRF, MoCA, NART, brain side of index lesion and WMH volume. Due to the explorative nature, these analyses were not corrected for multiple corrections and need to be interpreted with caution. As the size of our data did not allow the inclusion of all interaction terms in a single model, we repeated the model including all predictors and just one interaction term at a time and a random intercept for the individual participants. People who indicated they were ambidextrous (*n* = 2) were excluded from all analyses examining dexterity.

For visualization purposes only, we divided WMH volume change into quintiles. Quintile one reflects largest WMH decrease and quintile five reflects largest WMH volume increase.

SIS mobility and hand function scores were grouped to allow ordinal logistic regressions. SIS mobility was divided in three groups: (1) including maximum score of 45, reflecting participants who experience no difficulties at all; (2) scores 36 to 44, reflecting participants experiencing very few or some difficulties; (3) scores below 36, reflecting participants who experienced difficulties or who could not do activities at all. For the SIS hand domain the groups were as follows: (1) including scores of 25, i.e. no difficulties at all; (2) scores 24 to 20, i.e. some difficulties; (3) 19 or lower, very difficult to use hand or not able to use hand at all. To compare the objective in-person assessments with the self-reported questionnaires, we performed three ordinal logistic regressions, corrected for age and sex, at time of the 1-year visit. One regression was performed with the TUG and SIS mobility domain score, the other two with the dominant and non-dominant 9HPT outcomes and the SIS hand function domain score.

## Results

**Figure 2:**
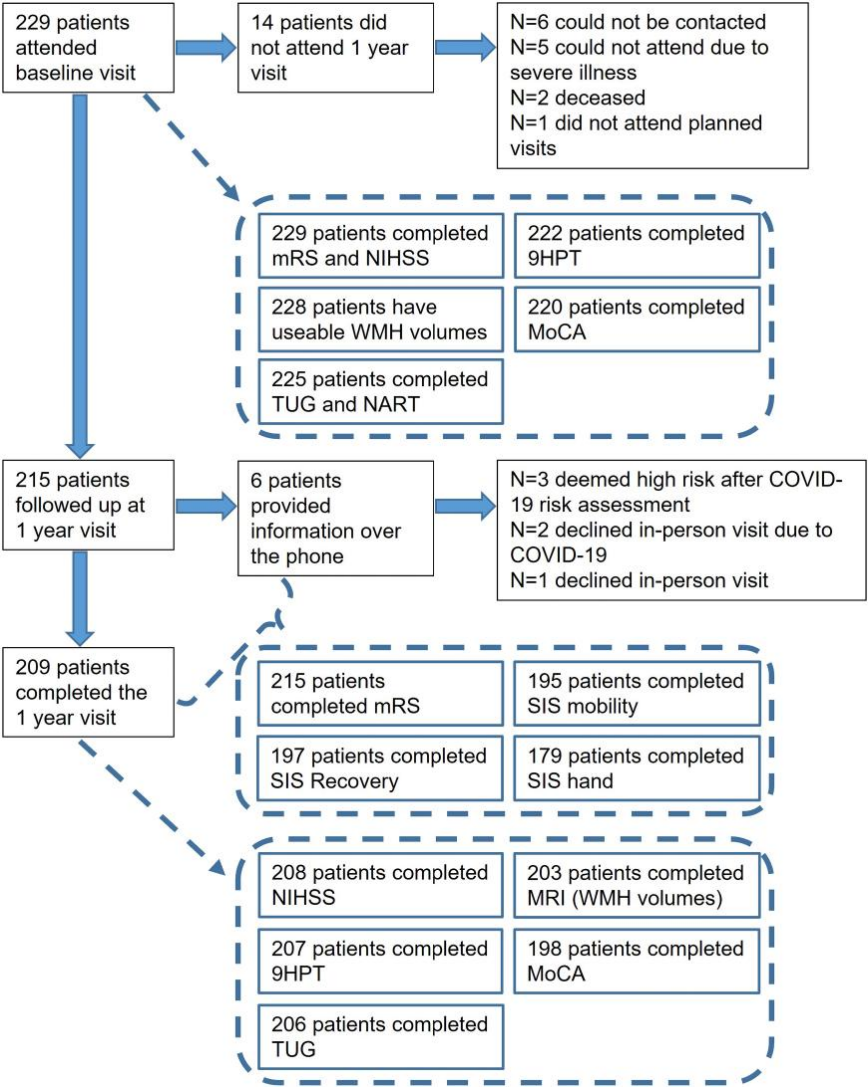
**Flow diagram of data collection at baseline and the 1 year visit.** Overview of the number of participants attending the baseline and 1 year visits, data completeness and reasons for missing data and non-attendance of the 1 year visit. 9HPT: 9 Hole Peg Test; MoCA: Montreal Cognitive Assessment; mRS: modified Rankin Scale; NART: National Adult Reading Test; NIHSS: National Institutes of Health Stroke Scale; SIS: Stroke Impact Scale; TUG: Timed Up and Go; WMH: White matter hyperintensities.

At baseline, 229 participants underwent MRI, the scan of one participant was not useable for analysis due to the quality of the scan (Fig. 2). The mean age at baseline was 65.9 years (standard deviation [SD] = 11.13), 66% were male, 57% had a subcortical stroke, a history of transient ischaemic attacks (TIA) or strokes prior to the index stroke that led to study inclusion was present in 16% (Table 1). There were slightly more index infarcts located in the right hemisphere (45.9%), than in the left hemisphere (40.6%). 66% of the participants had two or more vascular risk factors, the most common risk factors being hypercholesterolemia (75%) and hypertension (69%). At presentation, the median mRS score was 1 (interquartile range [IQR] 1 to 1), and the median NIHSS total score was 1 (IQR 0 to 2). The mean MoCA score was 25.05 (SD = 3.48), mean TUG time was 12.6 sec (SD = 6.28) and the mean dominant hand 9HPT time was 15.9 sec (SD = 5.78) and 17.7 sec for the non-dominant hand (SD = 6.87).

**Table 1:** Demographic characteristics of the mild stroke study 3 participants attending baseline and 1 year visit

	*N*	Baseline	*N*	1 year
Age, mean (SD)	229	65.85 (11.13)	215	66.79 (11.18)
Sex, male (%)	229	152 (66)	215	143 (62)
Index stroke subtype, *N* (%)	229		215	
Subcortical		130 (57)		124 (58)
Cortical		99 (43)		91 (42)
Brain side affected by index stroke, *n* (%)	229		215	
Right		105 (45.9)		100 (46.5)
Left		93 (40.6)		85 (39.5)
Both		16 (7.0)		16 (7.4)
Not visible		15 (6.6)		14 (6.5)
Handedness, *N* (%)	229		215	
Right		211 (92)		198 (92)
Left		16 (7)		15 (7)
Both		2 (1)		2 (1)
TIA or stroke before index stroke, yes (%)	229	36 (16)	-	-
TIA or stroke since baseline, yes (%)	-	-	228	13 (5.7)
Incidental DWI + lesion since stroke diagnosis, yes (%)	-	-	228	52 (22.8)
Hypertension, yes (%)	229	157 (69)	215	151 (70)
Hypercholesterolemia, yes (%)	229	171 (75)	215	160 (74)
History of smoking, yes (%)	229	44 (19)	215	35 (16)
Diabetes mellitus, yes (%)	229	50 (22)	215	47 (22)
VRF combined score (%)	229		215	
0		21 (9)		20 (9)
1		57 (25)		52 (24)
2		94 (41)		90 (42)
3		51 (22)		49 (23)
4		6 (3)		4 (2)
mRS, median (IQR), range	229	1 (1–1), 0–2	215	1 (0–1) 0–3
NIHSS, median (IQR), range	229	1 (0–2), 0–7	208	1 (0–2) 0–7
TUG time (seconds), mean (SD)	225	12.61 (6.28)	206	12.30 (5.51)
9HPT dominant hand (seconds), mean (SD)	222	15.90 (5.78)	207	16.70 (5.39)
9HPT non-dominant hand (seconds), mean (SD)	222	17.65 (6.87)	207	18.68 (8.16)
MoCA total score, mean (SD)	220	25.05 (3.48)	198	25.83 (3.57)
NART (total errors), mean (SD)	225	17.37 (9.53)	-	-
SIS—mobility (max. 45), median (IQR)	-	-	195	44 (38–45)
SIS—hand function (max. 25), median (IQR)	-	-	179	25 (21–25)
SIS—recovery (max. 100, i.e. full recovery), median (IQR)	-	-	197	90 (75–95)
Fazekas score periventricular WMH (%)	228		203	
0		8 (3.5)		11 (5.4)
1		111 (48.7)		91 (44.8)
2		59 (25.9)		55 (27.1)
3		50 (21.9)		46 (22.7)
Fazekas score deep WMH (%)	228		203	
0		21 (9.2)		20 (9.9)
1		122 (53.5)		110 (54.2)
2		59 (25.9)		45 (22.2)
3		26 (11.4)		28 (13.8)
WMH volume (ml), mean (SD)	228	14.93 (18.19)	203	16.55 (20.59)
WMH volume (%ICV), mean (SD)	228	0.934 (1.122)	203	1.032 (1.266)

9HPT, 9 hole peg test; DWI+, Diffusion weighted imaging positive; ICV, Intracranial volume; IQR, Interquartile range; MoCA, Montreal Cognitive Assessment; mRS, Modified Rankin Score; NART, National Adult Reading Test; NIHSS, National Institutes of Health Stroke Scale; SD, Standard Deviation; SIS, Stroke impact scale; TIA, Transient Ischaemic Attack; TUG, Timed-up and go; VRF, Vascular risk factors; WMH, White matter hyperintensity.

All participants were invited for the 1 year follow-up, 94% (*n* = 215) returned of whom six had a telephone follow-up (Fig. 2). Of the 14 participants who did not return, 2 were deceased, and 5 had a severe illness and could not attend an in-person or telephone study visit (Fig. 2). After comparison of baseline characteristics of participants who did and did not return for the 1-year visit, only the TUG time and dominant hand 9HPT time differed between the two groups (Supplementary Table 1). The people who did not attend their 1-year visit had faster TUG times and were slightly faster on the 9HPT.

At the 1-year visit, the prevalence of VRF (Table 1) was comparable to baseline as were mRS and NIHSS scores. Mean TUG time was 12.30 sec (SD = 5.51), mean 9HPT time was slightly longer than at baseline with 16.70 sec (SD = 5.39) for the dominant hand and 18.68 sec (SD = 8.16) for the non-dominant hand. Mean MoCA score was 25.83 (SD = 3.57). A total of 13/228 participants (5.7%) had had a clinical stroke or TIA since the baseline visit and 52/288 had an incidental diffusion-weighted positive lesion. Four participants had both.

Most WMH change in any direction, i.e. regression and progression, was seen in participants with most baseline WMH volumes (Supplementary Fig. 1).

### Mobility

The linear mixed model shows that, across a 1-year period, the TUG time increased with increasing age (standardized Β [standardized 95% confidence interval (CI)]: 0.124 [0.011 to 0.238], increasing NIHSS scores (0.106, [0.032 to 0.180]), increasing mRS (0.152 [0.073 to 0.231]) and increasing WMH volumes (0.176 [0.061 to 0.291]), Table 2. The change in TUG time was smaller for men than women (−0.306 [−0.533 to −0.078]). More VRF did not contribute to TUG time change, neither did stroke subtype (Supplementary Table 2).

**Table 2:** Results of linear mixed model assessing mobility with the timed-up and go

Predictors	Standardized Beta	Standardized 95% CI	*P* value
Age	0.124	0.011, 0.238	0.032
Sex (male)	−0.306	−0.533, −0.078	0.009
NIHSS	0.106	0.032, 0.180	0.005
mRS	0.152	0.073, 0.231	<0.001
Vascular risk factor (combined score)	0.027	−0.074, 0.129	0.599
WMH volume (%ICV)	0.176	0.061, 0.291	0.003

CI, Confidence Interval; ICV, Intracranial volume; mRS, modified Rankin Score; NIHSS, National Institutes of Health Stroke Scale; WMH, White matter hyperintensity.

**Figure 3:**
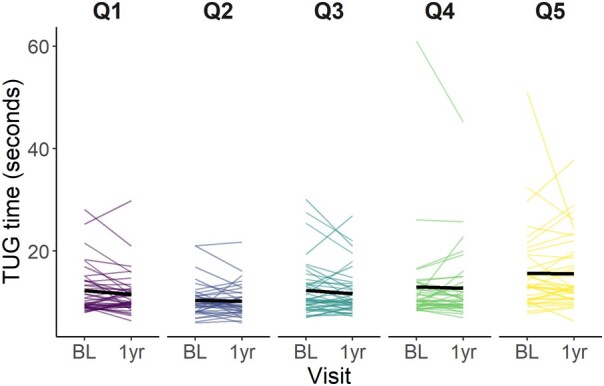
**Time on the timed-up and go test between baseline and 1-year visit per quintile of WMH volume (%ICV) change over 1 year.** Q1 is greatest WMH volume reduction and Q5 represents the greatest WMH increase. BL: Baseline; ICV: Intracranial volume; Q: Quintile; TUG: Timed- Up and Go; WMH: White matter hyperintensity.

In the 1-year period between baseline and follow-up, mean TUG time decreased slightly from 12.61 sec (*n* = 225; Table 1) to 12.30 sec (*n* = 206). TUG time change per quintile of WMH volume change between baseline and 1-year visit is plotted in Fig. 3. The figure suggests that the mean TUG time (black lines in Fig. 3) decreased slightly between visits in all quintiles, but times were generally longer in individuals with greater WMH volume increase.

### Dexterity

Based on the linear mixed models, increase in 9HPT time over 1 year was larger for the non-dominant hand (Table 3; 0.290 [0.155 to 0.424]) than the dominant hand. 9HPT time increase was associated with older age (0.102 [0.012 to 0.192]), male sex (0.182 [0.008 to 0.356]), increasing NIHSS (0.160 [0.094 to 0.226]), increasing mRS (0.100 [0.032 to 0.169]), decreasing MoCA score (−0.090 [−0.167 to −0.014]) and increasing WMH volume (0.104 [0.015 to 0.193]). The average time for the dominant hand was 16.1 sec (95% CI = 15.2 to 16.9) and for the non-dominant hand the mean time was 18.0 sec (95% CI = 17.0 to 18.9). The estimated difference between the hands was 1.88 sec (*t*(219) = −4.227, *P* < 0.001). Stroke subtype did not affect 9HPT time (Supplementary Table 3).

**Table 3:** Results linear mixed model assessing dexterity with the 9 hole peg test

	Standardized Beta	Standardized 95% CI	*P* value
Hand (non-dominant)	0.290	0.155, 0.424	<0.001
Age	0.102	0.012, 0.192	0.027
Sex (male)	0.182	0.008, 0.356	0.040
NIHSS	0.160	0.094, 0.226	<0.001
mRS	0.100	0.032, 0.169	0.004
Vascular risk factor (combined score)	−0.024	−0.105, 0.057	0.562
Side index lesion (both)	0.071	−0.260, 0.401	0.675
Side index lesion (not visible)	0.003	−0.338, 0.343	0.988
Side index lesion (right)	−0.017	−0.193, 0.158	0.845
NART (errors)	0.013	−0.076, 0.102	0.767
MoCA score	−0.090	−0.167, −0.014	0.021
WMH volume (%ICV)	0.104	0.015, 0.193	0.023

CI, Confidence Interval; ICV, Intracranial volume; MoCA, Montreal Cognitive Assessment; mRS, modified Rankin Score; NART, National Adult Reading Test; NIHSS, National Institutes of Health Stroke Scale; WMH, White matter hyperintensity.

**Figure 4:**
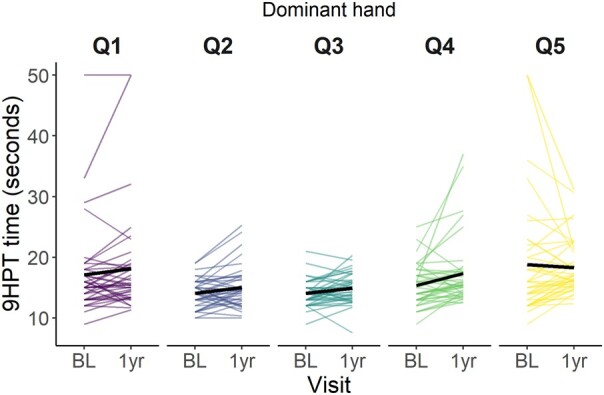
**Dominant hand 9 hole peg test time over baseline and 1-year visit per quintile of WMH volume (%ICV) change.** Q1 represents the greatest WMH volume reduction, Q5 the greatest WMH volume increase. 9HPT: 9 Hole Peg Test; BL: Baseline; ICV: Intracranial volume; Q: Quintile; WMH: White matter hyperintensity.

**Figure 5:**
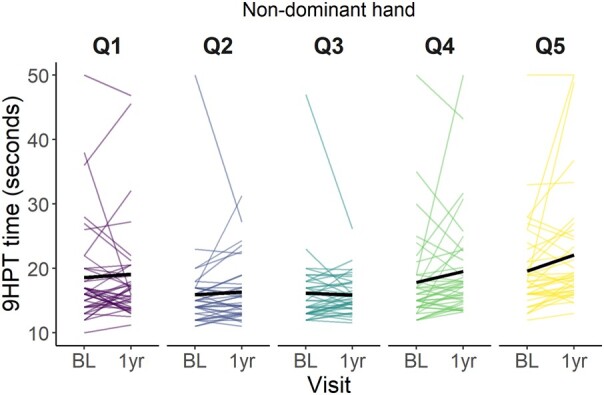
**Non-dominant hand 9 hole peg test time over baseline and 1-year visit per quintile of WMH volume (%ICV) change.** Q1 reflects the greatest WMH volume reduction, Q5 the greatest WMH volume increase. 9HPT: 9 Hole Peg Test; BL: Baseline; ICV: Intracranial volume; Q: Quintile; WMH: White matter hyperintensity.

In Figs 3 and 4 the times on the 9HPT are plotted at baseline and 1 year visit per quintile of WMH volume change for the dominant (Fig. 4) and non-dominant hand (Fig. 5). The plots show that for the dominant hand, the mean time on the 9HPT increases over time in all quintiles. In Q5, the mean time might be going down slightly. Mean time for the non-dominant hand also increases over time. Mean time in Q3 shows a slight decrease in time, however, this could be driven by the time of one participant.

We further explored the interaction between predictors and time for the dominant versus non-dominant hand using linear mixed models (Supplementary Tables 2–12). For the 9HPT, increasing NIHSS scores are associated with a greater increase in time for the non-dominant (0.188 [0.080 to 0.295]) than for the dominant hand (0.075 [−0.007 to 0.157]) (Supplementary Table 7). An index infarct in the right hemisphere is also related to increasing 9HPT time for the non-dominant hand (0.492 [0.207 to 0.777]) and time tends to decrease for the dominant hand (−0.191 [−0.393 to 0.011]) (Supplementary Table 9). Other predictors did not suggest an interaction with handedness.

### Objective in-person assessment versus self-reported experience via the stroke impact scale

The impact of stroke on mobility and hand function was self-reported with the SIS subdomains of mobility (*n* = 195) and hand function (*n* = 179) 1 year after stroke. Higher scores on the domains indicate less impact of the stroke on the specific domain. Overall, participants reported good mobility with a median score of 44/45 (IQR 38 to 45; Table 1). Based on logistic regression analysis, for men, the odds of higher scores on the SIS mobility domain were greater than for women (odds ratio [OR] 1.99 [95% CI 1.04 to 3.85]). For every one-second increase in TUG time, the odds of a lower SIS mobility score increased (OR 0.67 [0.60 to 0.75]). Age was not associated with SIS mobility (OR 0.99 [0.96 to 1.02]).

Participants reported good hand function, median 25/25 (IQR 21 to 25). Logistic regression shows that age was not associated with SIS hand domain scores, for either dominant (OR 1.0 [0.97 to 1.03]) or non-dominant hand (OR 0.99 [0.96 to 1.02]). For the dominant hand, the odds for men having a higher score on the SIS was greater than for women (OR 2.58 [1.36 to 4.94]). Per one-second increase on the 9HPT, the odds for a lower SIS hand function increased (OR 0.79 [0.71 to 0.86]). For the non-dominant hand, the odds of having a higher score on the SIS were greater for men versus women (OR 2.86 [1.52 to 5.42]). Per one-second increase on the 9HPT with the non-dominant hand, the odds for a lower score on the SIS hand domain increased (OR 0.88 [0.83 to 0.93]).

At 1 year, participants (*n* = 197) reported that they were close to full recovery (median = 95, IQR 75 to 95; Table 1). However, none of the participants reported that they were fully recovered from their stroke.

## Discussion

Our longitudinal observational study of people with lacunar or minor cortical ischaemic stroke and features of SVD shows that a decrease in mobility and dexterity is associated with WMH volume increase between baseline and 1 year, independent of stroke severity. A more severe stroke and an index lesion in the right hemisphere might affect hands differently and lead to worse dexterity of the non-dominant hand. In-person objective measurement of mobility relates closely with self-reported change in mobility after stroke, as are the measures of dexterity and self-reported hand function. However, based on self-reported measures, changes in function of the dominant hand might have a bigger impact on life than similar changes in the non-dominant hand, perhaps reflecting that patients are less likely to be affected by deficits in the non-dominant hand even when objective testing shows these to be present.

WMH volume increase contributes to worsening mobility, independently of other variables including sex, stroke severity and disability. This contribution of WMH to declining mobility was previously established in (older) people with sporadic SVD but without stroke.^4-8,10^ In stroke research, the contribution of WMH might be overlooked, as impaired mobility is often measured in relation to hemiparesis or hemiplegia, which suggests a more severe stroke. WMH might not be taken into account when assessing long-term outcomes. Some people with a minor stroke might experience weakness, but it might be deemed not severe or it improves. While WMH presence and changes are more examined in minor stroke, mobility might not be as well examined as in severe stroke. Some cross-sectional studies with small sample sizes, *n* = 12^53^ and *n* = 82,^54^ found that patients with minor stroke or TIA can have worse mobility than healthy controls. These findings were supported by the Rotterdam Study^55^ where gait of 147 community-dwelling participants with a prior ischaemic stroke was compared to gait of stroke-free participants. While the stroke participants did not report any gait problems at time of the stroke, they did have worse gait than the stroke-free participants. WMH and stroke might contemporaneously affect gait and mobility as stroke and sporadic SVD features, such as WMH, disrupt diffuse white matter connections and can affect mobility in the long term.^55-58^

In our longitudinal analysis, decline in dexterity was more strongly associated with the non-dominant hand compared to the dominant hand, and also with stroke severity, male sex compared to female sex, WMH volume increase during follow-up, older age, worse disability and declining global cognition. Current literature mainly describes cross-sectional associations between dexterity and WMH. WMH were not found to be associated with worse dexterity among population-based adults (mean age 57.2 years).^10^ However, in healthy adults with a higher risk of vascular disease^13^ and with sporadic SVD,^59^ WMH were cross-sectionally associated with worse dexterity. The finding in our longitudinal study that WMH can independently affect dexterity, builds on previous literature evidence that WMH might play a role in extremity motor deficits due to stroke (*n* = 28).^23^ While it might be expected that the hand contralateral to the index lesion is worst affected, a small cross-sectional study examining 30 haemorrhagic and ischaemic stroke patients found that dexterity was worst affected in the ipsilateral hand.^15^ In healthy right-handed participants (*n* = 44), worse fine motor movements with the non-dominant hand were associated with worse WMH cross-sectionally.^60^ Again, the dual effects of stroke (lesion) and WMH in minor stroke have not been widely examined in relation to dexterity. When we explored the difference in dexterity of the dominant and non-dominant hand, we found that an index lesion in the right hemisphere leads to worse dexterity of the non-dominant hand 1 year after experiencing the stroke (Supplementary Table 9). This is likely due to 46% of the participants having a right hemisphere lesion and the left hand being the non-dominant hand for 92% of the participants. However, the difference in effect between dominant and non-dominant hand is small. A more severe stroke was also predictive of worse dexterity. This effect was bigger for the non-dominant than the dominant hand. The non-dominant hand might potentially be more sensitive to any disruptions of white matter networks as it is less well trained than the dominant hand.^59-61^ Left-right differences and white matter networks involved in dexterity is beyond the scope of this paper and would require further research.

In addition to quantitative measures, we did collect self-reported hand function of the hand most affected by stroke, and mobility at 1 year after stroke. We found that the in-person measures and self-reported measures using the SIS are associated. Interestingly, the in-person measures suggest that the non-dominant hand performs worse on the 9HPT than the dominant hand and is more affected by stroke severity. However, the self-reported scores indicate that a worse performance on the in-person objective test (9HPT) for the dominant hand is more related to experienced difficulties in hand function, as reported on the questionnaire, than for the non-dominant hand. This could be explained by the fact that the dominant hand is used most of the time and for more complex fine motor movements and therefore changes are more likely to be noticed than in the non-dominant hand. This points out the importance of the experience of patients in relation to what we can measure. Lai and colleagues^62^ found that patients (*n* = 81) who were considered to be recovered 90 days after stroke, still could experience an impact of their stroke on their daily living, physical function, participation in life, and hand function when asked about experience via a questionnaire. Previously, we found among a similar population of minor stroke patients to our current study, that hand function and mobility can still be negatively affected 3 years after stroke.^63^ This shows that patients with minor stroke with mild symptoms can still experience (physical) difficulties, even when they are considered to be recovered 3 months after stroke,^62^ a year after stroke^64^ and even after 3 years.^63^ These difficulties can still affect their lives^65^ and our current results suggest that this might be worsened by the presence and progression of WMH.

The strengths of this study are the longitudinal nature of the study, the large sample size and inclusion of minor stroke patients, with 94% of the participants providing data at the 1-year follow-up visit. There is a gap in the literature on mobility and dexterity outcomes after minor stroke in general and particularly in combination with the presence and progression of SVD features which are common in patients with stroke. This study combines this information and not only corrects for WMH but also examines the role of WMH. We also combined data from left- and right-handed people by looking at dominant and non-dominant hands, instead of including only right-handed participants, since the latter limits data and does not represent the general population. Additionally, we take into account how the participants experience their mobility and hand function. This provides valuable information on not just the effect of minor stroke and SVD but indirectly reflects their recovery and raises awareness of potential clinical needs after a minor stroke. However, our explorative analyses on the effects of several factors on dexterity and hand dominance need to be interpreted with caution. Due to the sample size, we were not able to include all interactions in one model and due to the explorative nature of the analyses, no correction for multiple comparisons has been done. The explorative analyses might still be underpowered due to inclusion of the interactions. We did not include the time interval between stroke and baseline visit, which might have an influence on the baseline scores. It might be valuable to include this in future analyses.

Some participants were not able to or chose not to attend the 1-year follow-up visit. Six participants did not attend in person and we gathered their 1-year information via a telephone follow-up. We compared participants (*n* = 14) without a 1-year visit to participants with an in-person or telephone visit and we only found that they had a shorter TUG time and potentially shorter time on the 9HPT for the dominant hand. There is no clear explanation for this. Perhaps they were not as severely affected by their stroke, or perhaps they were fully recovered and were less inclined to continue. There is no significant difference in baseline NIHSS score between the 1 year attendees and non-attendees, but the IQR (0 to 1) and range (0 to 4) of the baseline NIHSS score might suggest that the non-attendees included people with lower stroke severities than the attendee group (baseline IQR = 0 to 2; range: 0 to 7).

We would encourage further studies on the influence of the presence and progression of all SVD features on mobility, dexterity and functional outcomes after minor ischaemic stroke. As all SVD features, not only WMH, might have a contemporaneous effect on functioning and recovery after stroke. This can be an opportunity to highlight recovery and potential aspects for clinical support and rehabilitation that might currently be overlooked. Mild symptoms at the time of stroke might not necessarily indicate potential for full recovery and can still have an impact on patient’s independence and daily living.

Our findings show that WMH changes are independently associated with worsening mobility and dexterity up to 1 year after minor ischaemic stroke. Non-dominant hand dexterity seems to be affected more by stroke than the dominant hand, but effects of the stroke on the dominant hand appear to have a bigger impact on patients’ daily lives. Measures of mobility and dexterity at 1 year post-stroke are associated with self-reported experiences of mobility and hand function difficulties. Self-reported patient data offers valuable additional information. It also shows that no symptoms to mild symptoms after a minor stroke can still result in difficulties 1 year later, sustained by WMH progression and incidental lesions.

## Supplementary Material

fcae133_Supplementary_Data

## Data Availability

Examples of the statistical models used can be found in the Supplementary material. Pipelines for WMH segmentation are publicly available.^43^ Details on the observational cohort study are published elsewhere.^24^ The data that we used in this study can be made available upon reasonable request to the corresponding author.
