# Supplementary material for: Impact of long-term white matter hyperintensity changes on mobility and dexterity
Source: Brain Commun. 2024 May 7;6(3):fcae133. doi: 10.1093/braincomms/fcae133 (PMC11074793; doi:10.1093/braincomms/fcae133)
Supplement: fcae133_Supplementary_Data [file fcae133_supplementary_data.docx]

**Supplementary material**

**Statistical models main analyses**

model1<- lmer(TUG ~ Age + Sex + NIHSS + mRS+ VRF + WMH volume + (1|Study ID), data=dataset)

model2 <- lmer(9HPT ~ Hand + Age + Sex + NIHSS + mRS + VRF + Side Index Lesion + baseline NART errors+ MoCA + WMH volume + (Hand | Study ID), data=dataset)

Hand: dominant vs non-dominant hand

NIHSS: National Institutes of Health Stroke Scale

mRS: modified Rankin Score

NART: National Adult Reading Test

MoCA: Montreal cognitive assessment

VRF: Vascular Risk Factors

WMH: White matter hyperintensity


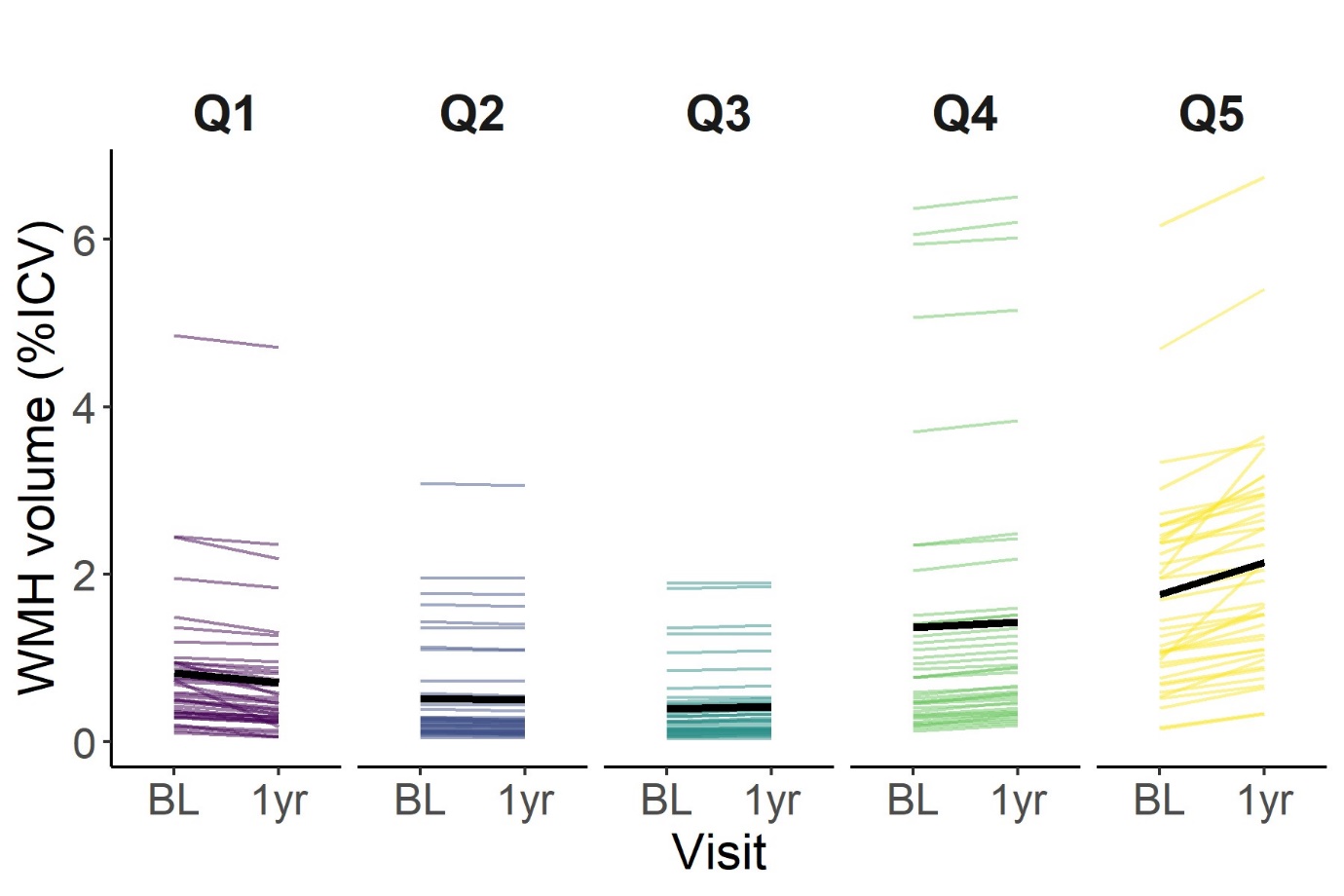


**Supplementary Figure 1. White matter hyperintensity (WMH) volume (% intracranial volume [ICV]) at baseline (BL) and 1 year visit per quintile (Q) of WMH volume change**.

**Supplementary Table 1.** Comparison of baseline characteristics participants who did and did not attend, either in person or via telephone, the 1-year follow-up visit.

|  | | | | **N** | **Follow-up attendees** | **N** | **Follow-up non-attendees** | **P value** |
| --- | --- | --- | --- | --- | --- | --- | --- | --- |
| Age, mean (SD) | | | | 215 | 65.74 (11.18) | 14 | 67.63 (10.61) | 0.529 |
| Sex, male (%) | | | | 215 | 143 (66.5) | 14 | 9 (64.3) | 1 |
| Stroke subtype (%) | | | | 215 |  | 14 |  | 0.420 |
|  | | Subcortical | |  | 124 (57.7) |  | 6 (42.9) |  |
|  | | Cortical | |  | 91 (42.3) |  | 8 (57.1) |  |
| Brain side affected by index stroke | | | | 215 |  | 14 |  | 0.579 |
|  | | | Right |  | 100 (46.5) |  | 5 (35.7) |  |
|  | | | Left |  | 85 (39.5) |  | 8 (57.1) |  |
|  | | | Both |  | 16 (7.4) |  | 0 (0) |  |
|  | | | Not visible |  | 14 (6.5) |  | 1 (7.1) |  |
| Handedness (%) | | | | 215 |  | 14 |  | 1 |
|  | | Right | |  | 198 (92.1) |  | 13 (92.9) |  |
|  | | Left | |  | 15 (7.0) |  | 1 (7.1) |  |
|  | | Both | |  | 2 (0.9) |  | 0 (0) |  |
| Previous TIA or stroke, yes (%) | | | | 215 | 33 (15.3) | 14 | 3 (21.4) | 0.553 |
| Hypertension, yes (%) | | | | 215 | 147 (68.4) | 14 | 10 (71.4) | 1 |
| Hypercholesterolemia, yes (%) | | | | 215 | 158 (73.5) | 14 | 13 (92.9) | 0.200 |
| History of smoking, yes (%) | | | | 215 | 41 (19.1) | 14 | 3 (21.4) | 0.736 |
| Diabetes mellitus, yes (%) | | | | 215 | 46 (21.4) | 14 | 4 (28.6) | 0.512 |
| VRF combined score (%) | | | | 215 |  | 14 |  | 0.682 |
|  | | 0 | |  | 21 (9.8) |  | 0 (0) |  |
|  | | 1 | |  | 54 (25.1) |  | 3 (21.4) |  |
|  | | 2 | |  | 88 (40.9) |  | 6 (42.9) |  |
|  | | 3 | |  | 46 (21.4) |  | 5 (35.7) |  |
|  | | 4 | |  | 6 (2.8) |  | 0 (0) |  |
| mRS, median (IQR), range | | | | 215 | 2 (2-2), 1-3 | 14 | 2 (2-2), 1-3 | 0.399 |
| NIHSS | | | | 215 | 1 (0-2) 0-7 | 14 | 1 (0-1) 0-4 | 0.197 |
| TUG time, seconds. Mean (SD) | | | | 212 | 12.76 (6.44) | 14 | 10.37 (1.74) | <0.001 |
| 9HPT dominant hand | | | | 208 | 15.99 (5.94) | 14 | 14.57 (2.14) | 0.054 |
| 9HPT non-dominant hand | | | | 208 | 17.67 (7.05) | 14 | 17.29 (3.20) | 0.698 |
| MoCA total score | | | | 208 | 25.03 (3.53) | 12 | 25.42 (2.61) | 0.632 |
| NART (total errors) | | | | 212 | 17.39 (9.61) | 13 | 17.08 (8.53) | 0.900 |
| Fazekas score periventricular WMH (%) | | | | 214 |  | 14 |  | 0.175 |
|  | 0 | | |  | 8 |  | 0 |  |
|  | 1 | | |  | 100 |  | 11 |  |
|  | 2 | | |  | 58 |  | 1 |  |
|  | 3 | | |  | 48 |  | 2 |  |
| Fazekas score deep WMH (%) | | | | 214 |  | 14 |  | 0.246 |
|  | 0 | | |  | 19 |  | 2 |  |
|  | 1 | | |  | 112 |  | 10 |  |
|  | 2 | | |  | 58 |  | 1 |  |
|  | 3 | | |  | 25 |  | 1 |  |
| WMH volume (ml) | | | | 214 | 15316.4 (18618.19) | 14 | 9024.0 (7582.0) | 0.352 |
| WMH volume (%ICV) | | | | 214 | 0.9568 (1.1474) | 14 | 0.5819 (0.5253) | 0.356 |

Analyses performed for continuous data were the Welch’s t-test or Mann-Whitney U test when assumptions were not met. For categorical data a two-sided Fisher’s exact test was used due to the sample size difference between the groups. 9HPT: 9 Hole Peg Test; ICV: Intracranial volume; IQR: Interquartile range; MoCA: Montreal Cognitive Assessment; mRS: Modified Rankin Score; NART: National Adult Reading Test; NIHSS: National Institutes of Health Stroke Scale; SD: Standard deviation; TIA: Transient Ischemic Attack; TUG: Timed-Up and Go; VRF: Vascular Risk Factors; WMH: White matter hyperintensity

**Supplementary Table 2.** Results of additional linear mixed model assessing mobility with the timed-up and go including stroke subtype

| **Predictors** | **Standardised Beta** | **Standardised 95% CI** | **P value** |
| --- | --- | --- | --- |
| Age | 0.126 | 0.011, 0.241 | 0.032 |
| Sex (male) | -0.307 | -0.535, -0.078 | 0.009 |
| NIHSS | 0.106 | 0.032, 0.180 | 0.005 |
| mRS | 0.151 | 0.072, 0.231 | <0.001 |
| Vascular risk factor (combined score) | 0.025 | -0.079, 0.129 | 0.638 |
| Stroke subtype (cortical) | -0.020 | -0.242, 0.202 | 0.860 |
| WMH volume (%ICV) | 0.175 | 0.060, 0.291 | 0.003 |

CI: Confidence Interval; ICV: Intracranial volume; mRS: modified Rankin Score; NIHSS: National Institutes of Health Stroke Scale; WMH: White matter hyperintensity

**Supplementary Table 3.** Results additional linear mixed model assessing dexterity with the 9 hole peg test including stroke subtype

|  | **Standardised Beta** | **Standardised 95% CI** | **P value** |
| --- | --- | --- | --- |
| Hand (non-dominant) | 0.290 | 0.155 – 0.424 | <0.001 |
| Age | 0.104 | 0.012 – 0.195 | 0.027 |
| Sex (male) | 0.181 | 0.007 – 0.355 | 0.042 |
| NIHSS | 0.159 | 0.093 – 0.225 | <0.001 |
| mRS | 0.099 | 0.031 – 0.168 | 0.005 |
| Vascular risk factor (combined score) | -0.026 | -0.110 – 0.058 | 0.544 |
| Side index lesion (both) | 0.071 | -0.260 – 0.402 | 0.674 |
| Side index lesion (not visible) | 0.003 | -0.339 – 0.345 | 0.985 |
| Side index lesion (right) | -0.017 | -0.193 – 0.159 | 0.849 |
| NART (errors) | 0.014 | -0.075 – 0.104 | 0.752 |
| MoCA score | -0.090 | -0.167 – -0.014 | 0.021 |
| Stroke subtype (cortical) | -0.017 | -0.189 – 0.156 | 0.851 |
| WMH volume (%ICV) | 0.104 | 0.014 – 0.193 | 0.024 |

CI: Confidence Interval; ICV: Intracranial volume; MoCA: Montreal Cognitive Assessment; mRS: modified Rankin Score; NART: National Adult Reading Test; NIHSS: National Institutes of Health Stroke Scale; WMH: White matter hyperintensity

**Explorative dexterity linear mixed models with the 9HPT as outcome**

**Supplementary Table 4.** Explorative dexterity linear mixed model with added interaction between age and hand (dominant versus non-dominant)

|  | **Standardised Beta** | **Standardised 95% CI** | **P value** |
| --- | --- | --- | --- |
| Hand (non-dominant) | 0.290 | 0.155, 0.424 | <0.001 |
| Age | 0.093 | -0.009, 0.196 | 0.073 |
| Sex (male) | 0.182 | 0.008, 0.356 | 0.040 |
| NIHSS | 0.160 | 0.094, 0.226 | <0.001 |
| mRS | 0.100 | 0.032, 0.169 | 0.004 |
| VRF | -0.024 | -0.105, 0.057 | 0.563 |
| Side index lesion (both) | 0.071 | -0.260, 0.401 | 0.675 |
| Side index lesion  (not visible) | 0.002 | -0.338, 0.343 | 0.989 |
| Side index lesion (right) | -.0.018 | -0.193, 0.158 | 0.843 |
| NART (errors) | 0.013 | -0.076, 0.102 | 0.768 |
| MoCA score | -0.090 | -0.167, -0.014 | 0.021 |
| WMH volume (%ICV) | 0.104 | 0.015, 0.193 | 0.023 |
| Age * hand  (non-dominant) | 0.024 | -0.110, 0.158 | 0.723 |

CI: Confidence Interval; ICV: Intracranial volume; MoCA: Montreal Cognitive Assessment; mRS: modified Rankin Score; NART: National Adult Reading Test; NIHSS: National Institutes of Health Stroke Scale; VRF: Vascular risk factors; WMH: White matter hyperintensity

**Supplementary Table 5.** Explorative dexterity linear mixed model with added interaction between sex and hand (dominant versus non-dominant)

|  | **Standardised Beta** | **Standardised 95% CI** | **P value** |
| --- | --- | --- | --- |
| Hand (non-dominant) | 0.180 | -0.051, 0.411 | 0.126 |
| Age | 0.102 | 0.012, 0.193 | 0.027 |
| Sex (male) | 0.124 | -0.076, 0.324 | 0.225 |
| NIHSS | 0.160 | 0.094, 0.226 | <0.001 |
| mRS | 0.100 | 0.031, 0.168 | 0.004 |
| VRF | -0.024 | -0.105, 0.057 | 0.563 |
| Side index lesion (both) | 0.071 | -0.260, 0.401 | 0.675 |
| Side index lesion  (not visible) | 0.003 | -0.338, 0.344 | 0.988 |
| Side index lesion (right) | -0.017 | -0.193, 0.159 | 0.849 |
| NART (errors) | 0.014 | -0.075, 0.103 | 0.764 |
| MoCA score | -0.090 | -0.166, -0.014 | 0.021 |
| WMH volume (%ICV) | 0.104 | 0.015, 0.193 | 0.023 |
| Sex (male) * hand  (non-dominant) | 0.166 | -0.118, 0.450 | 0.252 |

CI: Confidence Interval; ICV: Intracranial volume; MoCA: Montreal Cognitive Assessment; mRS: modified Rankin Score; NART: National Adult Reading Test; NIHSS: National Institutes of Health Stroke Scale; VRF: Vascular risk factors; WMH: White matter hyperintensity

**Supplementary Table 6.** Explorative dexterity linear mixed model with added interaction between mRS and hand (dominant versus non-dominant)

|  | **Standardised Beta** | **Standardised 95% CI** | **P value** |
| --- | --- | --- | --- |
| Hand (non-dominant) | 0.290 | 0.155, 0.424 | <0.001 |
| Age | 0.102 | 0.012, 0.193 | 0.027 |
| Sex (male) | 0.182 | 0.008, 0.356 | 0.040 |
| NIHSS | 0.160 | 0.094, 0.226 | <0.001 |
| mRS | 0.092 | 0.007, 0.176 | 0.033 |
| VRF | -0.024 | -0.105, 0.057 | 0.562 |
| Side index lesion (both) | 0.072 | -0.259, 0.402 | 0.670 |
| Side index lesion (not visible) | 0.004 | -0.338, 0.345 | 0.984 |
| Side index lesion (right) | -0.016 | -0.192, 0.160 | 0.857 |
| NART (errors) | 0.014 | -0.075, 0.103 | 0.759 |
| MoCA score | -0.090 | -0.166, -0.014 | 0.021 |
| WMH volume (%ICV) | 0.104 | 0.015, 0.194 | 0.022 |
| mRS * hand (non-dominant) | 0.019 | -0.093, 0.131 | 0.741 |

CI: Confidence Interval; ICV: Intracranial volume; MoCA: Montreal Cognitive Assessment; mRS: modified Rankin Score; NART: National Adult Reading Test; NIHSS: National Institutes of Health Stroke Scale; VRF: Vascular risk factors; WMH: White matter hyperintensity

**Supplementary Table 7.** Explorative dexterity linear mixed model with added interaction between NIHSS and hand (dominant versus non-dominant)

|  | **Standardised Beta** | **Standardised 95% CI** | **P value** |
| --- | --- | --- | --- |
| Hand (non-dominant) | 0.290 | 0.158, 0.422 | <0.001 |
| Age | 0.104 | 0.013, 0.195 | 0.025 |
| Sex (male) | 0.189 | 0.014, 0.364 | 0.034 |
| NIHSS | 0.075 | -0.007, 0.157 | 0.073 |
| mRS | 0.099 | 0.031, 0.168 | 0.005 |
| VRF | -0.024 | -0.105, 0.058 | 0.569 |
| Side index lesion (both) | 0.089 | -0.244, 0.422 | 0.600 |
| Side index lesion (not visible) | 0.020 | -0.323, 0.363 | 0.909 |
| Side index lesion (right) | -0.002 | -0.179, 0.175 | 0.981 |
| NART (errors) | 0.019 | -0.070, 0.109 | 0.672 |
| MoCA score | -0.089 | -0.165, -0.013 | 0.022 |
| WMH volume (%ICV) | 0.107 | 0.018, 0.197 | 0.019 |
| NIHSS * hand (non-dominant) | 0.188 | 0.080, 0.295 | 0.001 |

CI: Confidence Interval; ICV: Intracranial volume; MoCA: Montreal Cognitive Assessment; mRS: modified Rankin Score; NART: National Adult Reading Test; NIHSS: National Institutes of Health Stroke Scale; VRF: Vascular risk factors; WMH: White matter hyperintensity

**Supplementary Table 8.** Explorative dexterity linear mixed model with added interaction between VRF and hand (dominant versus non-dominant)

|  | **Standardised Beta** | **Standardised 95% CI** | **P value** |
| --- | --- | --- | --- |
| Hand (non-dominant) | 0.290 | 0.155, 0.425 | <0.001 |
| Age | 0.012 | 0.012, 0.192 | 0.027 |
| Sex (male) | 0.182 | 0.008, 0.356 | 0.040 |
| NIHSS | 0.160 | 0.094, 0.226 | <0.001 |
| mRS | 0.100 | 0.032, 0.169 | 0.004 |
| VRF | -0.018 | -0.112, 0.076 | 0.707 |
| Side index lesion (both) | 0.071 | -0.260, 0.401 | 0.675 |
| Side index lesion (not visible) | 0.003 | -0.338, 0.334 | 0.988 |
| Side index lesion (right) | -0.017 | -0.193, 0.158 | 0.846 |
| NART (errors) | 0.013 | -0.076, 0.102 | 0.767 |
| MoCA score | -0.090 | -0.166, -0.014 | 0.021 |
| WMH volume (%ICV) | 0.104 | 0.015, 0.193 | 0.023 |
| VRF * hand (non-dominant) | -0.016 | -0.148, 0.115 | 0.808 |

CI: Confidence Interval; ICV: Intracranial volume; MoCA: Montreal Cognitive Assessment; mRS: modified Rankin Score; NART: National Adult Reading Test; NIHSS: National Institutes of Health Stroke Scale; VRF: Vascular risk factors; WMH: White matter hyperintensity

**Supplementary Table 9.** Explorative dexterity linear mixed model with added interaction between side of index lesion and hand (dominant versus non-dominant)

|  | **Standardised Beta** | **Standardised 95% CI** | **P value** |
| --- | --- | --- | --- |
| Hand (non-dominant) | 0.003 | -0.205, 0.211 | 0.976 |
| Age | 0.102 | 0.011, 0.192 | 0.027 |
| Sex (male) | 0.182 | 0.009, 0.356 | 0.040 |
| NIHSS | 0.160 | 0.094, 0.226 | <0.001 |
| mRS | 0.100 | 0.032, 0.169 | 0.004 |
| VRF | -0.024 | -0.105, 0.057 | 0.559 |
| Side index lesion (both) | -0.054 | -0.431, 0.323 | 0.779 |
| Side index lesion (not visible) | -0.177 | -0.567, 0.214 | 0.375 |
| Side index lesion (right) | -0.191 | -0.393, 0.011 | 0.064 |
| NART (errors) | 0.013 | -0.076, 0.102 | 0.769 |
| MoCA score | -0.090 | -0.167, -0.014 | 0.020 |
| WMH volume (%ICV) | 0.104 | 0.014, 0.193 | 0.023 |
| Side index lesion (both)* hand (non-dominant) | 0.355 | -0.171, 0.881 | 0.186 |
| Side index lesion (not visible) * hand (non-dominant) | 0.510 | -0.035, 1.055 | 0.067 |
| Side index lesion (right) * hand (non-dominant) | 0.492 | 0.207, 0.777 | 0.001 |

CI: Confidence Interval; ICV: Intracranial volume; MoCA: Montreal Cognitive Assessment; mRS: modified Rankin Score; NART: National Adult Reading Test; NIHSS: National Institutes of Health Stroke Scale; VRF: Vascular risk factors; WMH: White matter hyperintensity

**Supplementary Table 10.** Explorative dexterity linear mixed model with added interaction between NART and hand (dominant versus non-dominant)

|  | **Standardised Beta** | **Standardised 95% CI** | **P value** |
| --- | --- | --- | --- |
| Hand (non-dominant) | 0.289 | 0.154, 0.423 | <0.001 |
| Age | 0.102 | 0.012, 0.192 | 0.027 |
| Sex (male) | 0.182 | 0.009, 0.356 | 0.040 |
| NIHSS | 0.160 | 0.094, 0.226 | <0.001 |
| mRS | 0.100 | 0.032, 0.169 | 0.004 |
| VRF | -0.024 | -0.105, 0.057 | 0.561 |
| Side index lesion (both) | 0.071 | -0.260, 0.401 | 0.675 |
| Side index lesion (not visible) | 0.002 | -0.339, 0.343 | 0.989 |
| Side index lesion (right) | -0.017 | -0.193, 0.158 | 0.847 |
| NART (errors) | -0.011 | -0.112, 0.090 | 0.834 |
| MoCA score | -0.090 | -0.167, -0.014 | 0.021 |
| WMH volume (%ICV) | 0.104 | 0.015, 0.193 | 0.023 |
| NART * hand (non-dominant) | 0.069 | -0.066, 0.203 | 0.315 |

CI: Confidence Interval; ICV: Intracranial volume; MoCA: Montreal Cognitive Assessment; mRS: modified Rankin Score; NART: National Adult Reading Test; NIHSS: National Institutes of Health Stroke Scale; VRF: Vascular risk factors; WMH: White matter hyperintensity

**Supplementary Table 11.** Explorative dexterity linear mixed model with added interaction between MoCA and hand (dominant versus non-dominant)

|  | **Standardised Beta** | **Standardised 95% CI** | **P value** |
| --- | --- | --- | --- |
| Hand (non-dominant) | 0.290 | 0.155, 0.424 | <0.001 |
| Age | 0.105 | 0.014, 0.195 | 0.024 |
| Sex (male) | 0.183 | 0.009, 0.357 | 0.039 |
| NIHSS | 0.160 | 0.095, 0.226 | <0.001 |
| mRS | 0.099 | 0.031, 0.168 | 0.004 |
| VRF | -0.024 | -0.105, 0.057 | 0.564 |
| Side index lesion (both) | 0.074 | -0.257, 0.406 | 0.659 |
| Side index lesion (not visible) | 0.007 | -0.335, 0.348 | 0.968 |
| Side index lesion (right) | -0.014 | -0.190, 0.162 | 0.874 |
| NART (errors) | 0.017 | -0.072, 0.107 | 0.701 |
| MoCA score | -0.057 | -0.149, 0.035 | 0.225 |
| WMH volume (%ICV) | 0.105 | 0.016, 0.195 | 0.021 |
| MoCA * hand (non-dominant) | -0.073 | -0.191, 0.044 | 0.220 |

CI: Confidence Interval; ICV: Intracranial volume; MoCA: Montreal Cognitive Assessment; mRS: modified Rankin Score; NART: National Adult Reading Test; NIHSS: National Institutes of Health Stroke Scale; VRF: Vascular risk factors; WMH: White matter hyperintensity

**Supplementary Table 12.** Explorative dexterity linear mixed model with added interaction between WMH volume and hand (dominant versus non-dominant)

|  | **Standardised Beta** | **Standardised 95% CI** | **P value** |
| --- | --- | --- | --- |
| Hand (non-dominant) | 0.290 | 0.155, 0.425 | <0.001 |
| Age | 0.102 | 0.012, 0.192 | 0.027 |
| Sex (male) | 0.181 | 0.008, 0.355 | 0.041 |
| NIHSS | 0.160 | 0.094, 0.226 | <0.001 |
| mRS | 0.100 | 0.031, 0.168 | 0.004 |
| VRF | -0.024 | -0.105, 0.057 | 0.566 |
| Side index lesion (both) | 0.070 | -0.261, 0.400 | 0.678 |
| Side index lesion (not visible) | 0.002 | -0.339, 0.343 | 0.991 |
| Side index lesion (right) | -0.019 | -0.194, 0.157 | 0.835 |
| NART (errors) | 0.013 | -0.076, 0.102 | 0.770 |
| MoCA score | -0.090 | -0.166, -0.014 | 0.021 |
| WMH volume (%ICV) | 0.080 | -0.022, 0.181 | 0.123 |
| WMH volume (%ICV) * hand (non-dominant) | 0.068 | -0.066, 0.203 | 0.318 |

CI: Confidence Interval; ICV: Intracranial volume; MoCA: Montreal Cognitive Assessment; mRS: modified Rankin Score; NART: National Adult Reading Test; NIHSS: National Institutes of Health Stroke Scale; VRF: Vascular risk factors; WMH: White matter hyperintensity
